# Lightweight and efficient neural network with SPSA attention for wheat ear detection

**DOI:** 10.7717/peerj-cs.931

**Published:** 2022-04-05

**Authors:** Yan Dong, Yundong Liu, Haonan Kang, Chunlei Li, Pengcheng Liu, Zhoufeng Liu

**Affiliations:** 1 School of Electronic and Information Engineering, Zhongyuan University of Technology, ZhengZhou, China; 2 Department of Statistics and Data Science, National University of Singapore, Singapore; 3 Department of Computer Science, University of York, York, United Kingdom

**Keywords:** Wheat ears, Deep neural networks, Lightweight, Polarized self-attention

## Abstract

Advancements in deep neural networks have made remarkable leap-forwards in crop detection. However, the detection of wheat ears is an important yet challenging task due to the complex background, dense targets, and overlaps between wheat ears. Currently, many detectors have made significant progress in improving detection accuracy. However, some of them are not able to make a good balance between computational cost and precision to meet the needs of deployment in real world. To address these issues, a lightweight and efficient wheat ear detector with Shuffle Polarized Self-Attention (SPSA) is proposed in this paper. Specifically, we first utilize a lightweight backbone network with asymmetric convolution for effective feature extraction. Next, SPSA attention is given to adaptively select focused positions and produce a more discriminative representation of the features. This strategy introduces polarized self-attention to spatial dimension and channel dimension and adopts Shuffle Units to combine those two types of attention mechanisms effectively. Finally, the TanhExp activation function is adopted to accelerate the inference speed and reduce the training time, and CIOU loss is used as the border regression loss function to enhance the detection ability of occlusion and overlaps between targets. Experimental results on the Global Wheat Head Detection dataset show that our method achieves superior detection performance compared with other state-of-the-art approaches.

## Introduction

Wheat is one of the most globally essential cereal crops. Maintaining a high yield of wheat has a positive effect on ensuring social stability and promoting economic development. The number of wheat ears is an important factor that affects the yield of wheat. Therefore, how to quickly and precisely identify each wheat ear and count the total number is the key to the prediction of crop growth in complex agricultural conditions. Traditional counting methods rely on field surveys, sampling, and weighing, which is inefficient and time-consuming. It is difficult to use these methods to accurately estimate the yield on a large area, which severely limits their application in seed screening and breeding, pest control, density estimation, gene trait expression, and field management (Li et al., 2021). Due to these reasons, the application of deep learning techniques in detection of wheat spike has received much attention in recent years.

The continuous development of information technology in agriculture has led to widespread use of image processing methods for wheat ears detection. Feature extraction of wheat images based on shape, color, or texture features, as wells as the detection and counting of wheat ears through machine learning methods have improved the efficiency of wheat yield estimation to a certain extent. For instance, Cointault, Journaux & Gouton (2008) designed an automatic field walking robot to obtain an image of wheat per unit area, and then identified the number of wheat ears by solely extracting the color and texture features from the images. Alharbi, Zhou & Wang (2018) extracted the conversion of the original wheat plant image data to color index of vegetation, so as to segment the wheat head detection area, and used the Gabor filter and K-means clustering algorithm to achieve wheat head detection. Nevertheless, image processing and shallow learning methods require a lot of experience and are not robust to the variable natural environment in the field.

Advances in computer vision technology provide technical support for the automatic detection of wheat ears. Wheat spike detection methods based on convolutional neural networks not only improve the efficiency of wheat head counting and reduce the workload of manual work in the field but also have considerable accuracy, which has become a research hotspot. Hasan et al. (2018) collected 335 images of 10 varieties at three different growth stages and built SPIKE dataset. Regional convolutional neural networks (RCNN) (Girshick et al., 2015) target detection method was utilized to realize correct detection of wheat spikes, and the average accuracy of the model reached 93.4%. Madec et al. (2019) used Faster R-CNN (Fast Region Convolutional Neural Network) (Ren et al., 2015) for ear detection, which showed good robustness for ear detection at the maturity stage. Wangli et al. (2021) proposed an improved wheat detection method based on the YOLOv4 (Bochkovskiy, Wang & Liao, 2020) model, and the average detection accuracy reached 78.99% on the self-built dataset. However, it should be noted that existing methods (Hasan et al., 2018; Madec et al., 2019; Wangli et al., 2021) for wheat ear detection with deep learning focus on how to improve the detection accuracy, but do not consider the impact of detection speed and model capacity on edge devices.

To address these issues, we propose a detector for wheat ear detection deployed in resource-limited equipment, namely, the lightweight and efficient wheat ear detection with SPSA attention (LE-SPSANet). Firstly, AsymmNet (Towards ultralight convolution neural networks using asymmetrical bottlenecks) (Yang, Shen & Zhao, 2021) is adopted as the backbone network in this study to reduce model capacity and complexity. Then, the detection accuracy of overlapping ears is improved by well-designed SPSA attention, which introduces Polarized self-attention to spatial dimension and channel dimension, and adopts Shuffle Units to combine two types of attention mechanisms effectively. Meanwhile, the TanhExp activation function is used to reduce training time and the capacity of the neural network. Finally, CIOU loss (Zheng et al., 2020) is adopted, which improves the convergence ability of the model, accelerates the training speed of the network model, and improves the regression accuracy of the prediction box. In conclusion, the contributions of this article are summarized as follows:

• An efficient and lightweight wheat ear detection method based on asymmetric bottleneck convolution and high convergence speed smooth activation function is proposed to realize fast and accurate automatic wheat ear detection.

• Shuffle Polarized Self-Attention (SPSA), an effective attention mechanism module, is well-designed to reduce the model capacity and accelerate the detection rate of the neural network.

• Experimental results on the Global Wheat Head Detection dataset show that the proposed method not only greatly reduces the model capacity and calculation cost, but also improves the detection speed with almost no loss of accuracy.

## Related work

### Wheat ear detection task

The segmentation of wheat ears and the shallow learning method are the most important methods for the recognition and detection of wheat ears. Firstly, the shape, texture, and color features of wheat ears are extracted manually from the original RGB image. Subsequently, the model is constructed by the classifier to achieve automatic recognition and detection of wheat ears. Zhou et al. (2018) trained the WSVM-Seg model based on the shallow learning method by preprocessing the selected samples from the wheat data. This approach achieved a detection accuracy rate of up to 82%. Based on image processing technology, Fernandez-Gallego et al. (2018) used filtering and maximization to detect and count the number of wheat ears in the collected wheat ear images. The recognition accuracy of the algorithm reaches 90%. However, the use of manual feature design requires a lot of experience, and the robustness to fields with different light and soil conditions is not sufficient to broaden its application to obtain generalized models. At the same time, the detection accuracy needs to be further improved.

With the rapid development of artificial intelligence and computer science, deep learning has become a hot research topic and is widely used in more and more fields. By applying deep learning techniques to the wheat ears detection task, suitable deep neural network models are selected for training, and the obtained data-trained models are used to automatically detect wheat ears, which can meet the requirements of wheat yield estimation. The application of automatic wheat ear detection technology based on the deep neural network can not only improve detection efficiency but also help promote the development and progress of high-tech agriculture. Relevant studies are successful in applying deep learning models in wheat ear detection. Based on the image processing and deep learning technology, the EfficientDet algorithm (Tan, Pang & Le, 2020) was used to detect the wheat ear image by Cao et al. (2020), the final accuracy rate reached 92.92% and the test time of the single sheet was 0.2 s. An improved YOLOv4 with CBAM (convolutional block attention module) (Woo et al., 2018) including spatial and channel attention model was proposed by Yang et al. (2021). This strategy could enhance the feature extraction capabilities of the network by adding receptive field modules. Xu et al. (2020) used the K-means clustering method for automatic segmentation of wheat ear images and sent the segmented image tags to the convolutional neural network model for training and testing. Although much progress (Cao et al., 2020; Yang et al., 2021; Xu et al., 2020) has been made, there are still problems such as low efficiency, insufficient robustness, and poor performance. Simultaneously, the above-mentioned deep learning-based detection methods often need to sacrifice accuracy to ensure the inference speed of the detector because of the limitation of hardware in practice. In order to achieve the trade off among the conflicting performance requirements (Han et al., 2020), combining lightweight neural networks with advanced optimal algorithms to the task of wheat ear detection has attracted continuing attention in the past few years.

### Object detection

The continuous improvement of computing power has made data-driven deep learning methods appear on the stage of history, bringing about a series of excellent algorithms. Deep learning-based methods show superior performance over traditional algorithms in many computer vision fields (Hu et al., 2021; Liu et al., 2021b; Teng et al., 2021; Zhao et al., 2021). The current mainstream object detection methods can be classified into Anchor-based methods and Anchor-free methods according to whether candidate regions are generated, with the main difference being whether target regression and classification are performed by predefined anchor frames.

Anchor-based detection algorithms preset anchor frames of different scales and proportions on feature map to accommodate targets of different shapes. Since the detection process of these algorithms are not the same, they can be subdivided into two categories. One is the two-stage detection algorithm based on candidate regions represented by the R-CNN series. The two-stage detection algorithm process consists of two steps: the first step is to select candidate regions and the second step is to classify and regress candidate regions. For example, in 2014, Ross B. Girshick proposed R-CNN (Girshick et al., 2014), and later proposed Fast R-CNN (Girshick, 2015), Faster R-CNN (Ren et al., 2015), Mask R-CNN (He et al., 2017) and so on. The other is the one-stage detection algorithm based on regression analysis represented by the YOLO (you only look once) and SSD (single shot multibox detector) series. For instance, in 2016, Redmon et al. (2016) proposed the YOLO network. In addition, in response to the low accuracy of YOLO network target detection, researchers have successively proposed SSD (Liu et al., 2016), YOLOv2 (Redmon & Farhadi, 2017), YOLOv3 (Redmon & Farhadi, 2018), YOLOv4 (Bochkovskiy, Wang & Liao, 2020), TPH-YOLOv5 (Zhu et al., 2021), DETR (Zhu et al., 2020), Scaled-YOLOv4 (Wang, Bochkovskiy & Liao, 2021), EfficientDet (Tan, Pang & Le, 2020) and so on. The single-step target detection algorithm, especially the YOLO series, is significantly faster than the two-step target detection algorithm. Although the accuracy of the single-step algorithms is slightly lower than the two-step ones, it is still within the acceptable range and is preferred in most engineering applications.

Anchor-free target detection algorithms are divided into two types. One is the Dense Prediction type represented by DenseBox. This type of method regards the central area of the object as the foreground, defines the positive sample, and then predicts the distance between the objects and the four sides of the box. For example, Zhu, He & Savvides (2019) added an anchor-free branch and an online feature selection mechanism to RetinaNet (Lin et al., 2017). This branch defined the central area of the object as a positive sample, and used its distance between the object and the four sides to locate it. Tian et al. (2019) defined all positions within the object frame as positive samples and then detected the object through four distance values and one center score. Liu, Liao & Hasan (2019) only defined the center point of the object as a positive sample and detected pedestrians through a fixed aspect ratio. Kong et al. (2020) defined the middle position of the object as a positive sample, and each position had four distances for detection. The other is the keypoint-based Detection type. This type of anchor-free method first locates the pre-defined or self-learned key points and then generates a frame to detect the object. For example, Zhou, Zhuo & Krahenbuhl (2019) detected the top, left, bottom, right, and center points of the object to generate the border of the object, and CornerNet (Law & Deng, 2018) detected the border of the object through the upper left corner and the lower right corner. The anchor-free algorithm gets rid of the computationally intensive problem caused by the use of anchors, but its detection accuracy is currently not ideal.

### Attention mechanism and its variants

Attention mechanisms are widely used in many computer vision areas such as image classification, object detection, instance segmentation, semantic segmentation, scene parsing, and action localization. The SE (Squeeze-and-Excitation) (Hu, Shen & Sun, 2018) attention module improves the expressive capability of the network by dynamically modulating the weight of each channel and recalibrating the features. The global average collection feature is used to calculate the channel directional attention, and it is worth noting that the spatial attention problem tends to be neglected. Simultaneously, the number of parameters will increase obviously due to the use of the full connection layer. CBAM attentional module cascades channel attention and spatial attention along two independent dimensions (channel and space) and obtains attentional graphs by means of global average pooling and maximum pooling. Then, the attentional graphs are multiplied by the input feature graphs for adaptive feature refinement. The channel attention module is mainly used to measure the importance of channels to facilitate the selection of valid channel information. The spatial attention module is used to highlight the feature information of the target location in the feature map. BAM (Bottleneck Attention Module) (Park et al., 2018) uses a parallel method to integrate spatial attention and channel attention. DANet (Dual Attention Network for Scene Segmentation) proposed by Fu et al. (2019) explored the cross-correlation between each position and each channel in the input feature map to generate global spatial attention and channel attention respectively, which has a significant improvement in semantic segmentation tasks. The SK (Selective Kernel) structure proposed by Li et al. (2019) combines the idea of SE with the residual network, allowing the network to dynamically select different receptive fields according to the different scales of the feature map, further expanding the research on the attention mechanism. Although these attention models improve the feature expression capabilities of CNNs from different perspectives, they also add a lot of workload to the network. In this paper, we apply channel and spatial attention modules in parallel in LE-SPSANet, so that each module can learn what and where to focus on the channel and spatial axes, respectively. Different from the attention module in Woo et al. (2018) and Park et al. (2018), we introduce polarized self-attention (Liu et al., 2021a) in the spatial dimension and channel dimension, and use Shuffle Units to effectively combine the two attention mechanisms. This strategy improves detection accuracy while reducing model capacity.

## Proposed method

### Overall network structure

**Figure 1:**
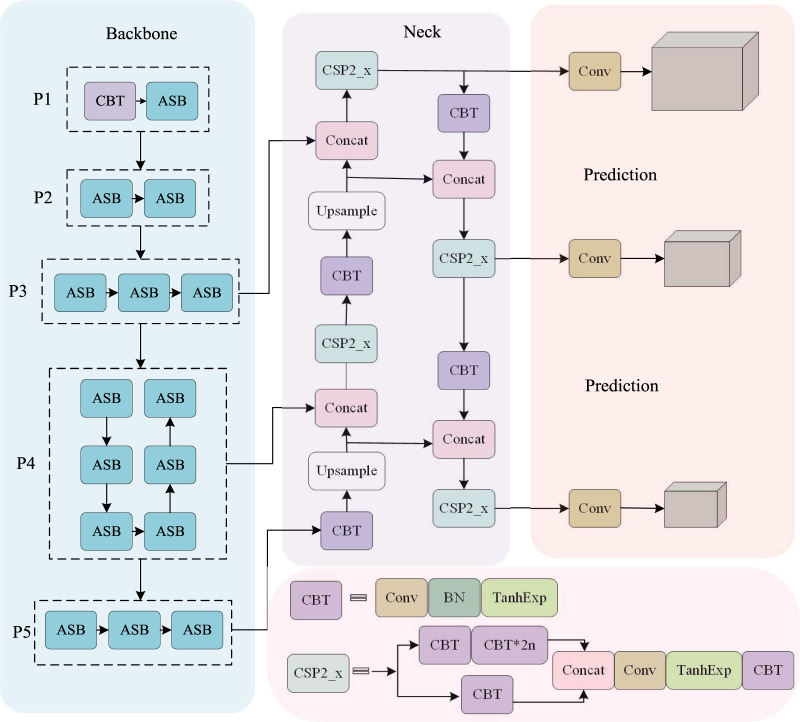
The overall structure of LE-SPSANet: (A) Asymmetric backbone with SPSA attention; (B) the neck use the structure like PANet; (C) three YOLO detection heads use the feature maps from encoder blocks in neck; (D) ASB and TanhExp components are provided in Fig. 2C and Eq. (1), respectively.

The network structure designed in this study is shown in Fig. 1, which consists of a backbone network, a detection neck, and three detection heads for object classification and localization. Specifically, we first use the refined AsymmNet with SPSA attention as the backbone network to extract features, which generates three effective feature layers P3, P4, and P5 to detect small, medium, and large-scale wheat targets, respectively. The neck is designed to make better use of the features obtained. It reprocesses and rationally uses the feature maps extracted by backbone at different stages. Then, we utilize top-down path-aggregation blocks to pass down strong semantic features from higher levels and utilize bottom-up pyramids to pass up strong localization features from lower levels. This strategy not only enhances the interfusion between different feature layers but also strengthens the semantic representation and localization capabilities of the feature graph at multiple scales. Finally, with overlap rate, scale, and penalty factor considered, the CIOU boundary regression loss function is used to further improve the detection performance on occluded and overlapped targets.

**Figure 2:**
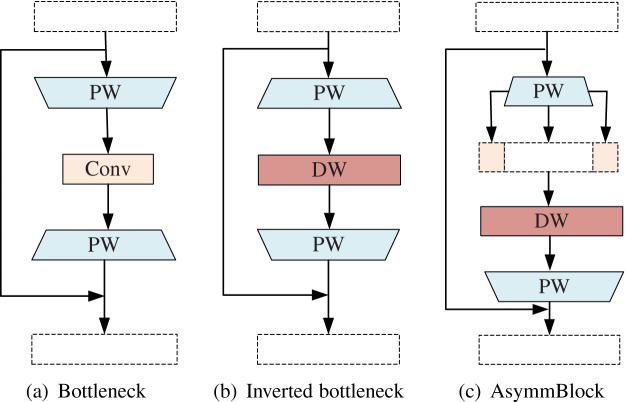
Different types of basic convolution blocks. (A) Bottleneck. (B) Inverted bottleneck. (C) AsymmBlock.

### Backbone network

AsymmNet is an efficient neural network model designed for computing and power-constrained devices. The model has the characteristics of small capacity and high precision. The detection of wheat ears could be affected by weed, wheat leaves and other factors in some complicated scenarios, making the background texture too complex to recognize. Considering the existing difficulties in wheat ear detection, this paper adopts the refined AsymmNet as the backbone network for feature extraction. The specific network structure is shown in Table 1, where the fourth column represents the number of channels after the AsymmBottleneck (ASB) internal inverse residual structure rises, the fifth column represents the number of channels input to the ASB feature layer, and the sixth column represents whether to join the SPSA attention mechanism. The network consists of 15 ASB modules which contain Depthwise Separable Convolution, TanhExp activation function (Liu & Di, 2020) and SPSA attention mechanism to improve its representation ability.

**Table 1:** Backbone network structure.

Stage	Input	Type	Exp size	Out	SPSA	Stride
	1024*1024*3	conv2d	–	16	–	2
P1	512*512*16	ASB, 3*3	16	16	–	1
	512*512*16	ASB, 3*3	64	24	–	2
P2	256*256*24	ASB, 3*3	72	24	–	1
	256*256*24	ASB, 5*5	72	40	+	2
	128*128*40	ASB, 5*5	120	40	+	1
P3	128*128*40	ASB, 5*5	120	40	+	1
	128*128*40	ASB, 3*3	240	80	–	2
	64*64*80	ASB, 3*3	200	80	–	1
	64*64*80	ASB, 3*3	184	80	–	1
	64*64*80	ASB, 3*3	184	80	–	1
	64*64*80	ASB, 3*3	480	112	+	1
P4	64*64*112	ASB, 3*3	672	112	+	1
	32*32*112	ASB, 5*5	672	160	+	2
	32*32*160	ASB, 5*5	960	160	+	1
P5	32*32*160	ASB, 5*5	960	160	+	1

In order to perform multi-scale feature fusion, the output layer of the 6th ASB module is taken as the P3 layer, the output of the 13th ASB module is taken as the P4 layer, and the output of the 15th ASB module is taken as the P5 layer. After upsampling, low-level features with more spatial details are channel-cascaded with high-level features, and the features of different channels are merged by 1 × 1 convolution. Due to the large variations in the scale of wheat ears, using only a single-scale feature layer cannot deal with targets of different scales at the same time. Based on the multi-scale detection strategy of YOLOv3, three layers are constructed after the fusion of P3, P4, and P5 for the detection of the large, medium, and small targets of different scales to improve accuracy and robustness. The asymmetric convolutional backbone network makes the model more lightweight and the TanhExp activation function speeds up the network training time. Use of the strategies mentioned all contribute significantly to the excellent performance of LE-SPSANet.

#### AsymmBottleneck

Deep Convolutional Neural Network (CNN) has achieved amazing results in wheat ear detection. However, due to limited memory and computing resources, it is hard to use these models on mobile or embedded devices. A compact network with fewer parameters and calculations to obtain good accuracy has become the main solution to the above problems. Although the compact network method adopts full-precision floating-point number as weights, it reduces total parameter and operations through a compact architecture design and minimize the loss of precision. Commonly used techniques include replacing most of the 3 × 3 filters with 1 × 1 filters. In addition, deep separable convolutions, channel transformations, and group convolutions can also be used to reduce operations.

Among them, the depth separable convolution was proposed by Chollet in the Xception network (Chollet, 2017). It is a combination of a depthwise (DW) convolution and a 1 × 1 pointwise (PW) convolution. DW convolution uses a single-channel filter to learn the difference between the positions in each channel. PW convolution learns new features by calculating the linear combination of all input channels. MobileNet series (V1–V3) (Howard et al., 2017; Sandler et al., 2018; Howard et al., 2019) is the most successful lightweight CNN model based on deep separable convolution and intelligent architecture design so far, which is widely used in multiple computer vision tasks.

Sandler et al. (2018) proposed a reverse bottleneck for MobileNetV2. Different from the original bottleneck design (2(a)), the reverse bottleneck block (2(b)) uses a low-dimensional (number of input channels) input tensor and expands it into a high-dimensional tensor through pointwise convolution quantity. The expanded high-dimensional tensor is sent to the deep separable convolution, and the corresponding point-wise convolution is subjected to deep convolution. After that, low-dimensional new features are generated through the linear combination channel. It can be seen that the first point-to-convolution expands the information flow and increases the capacity, and the subsequent convolution operator is responsible for the expression of this layer.

However, AsymmNet believed that the two PW convolutions contribute differently in the original inverted residual bottleneck block. The first PW convolution is mainly used to expand the channel, the second DW convolution is used to learn the spatial correlation of features, and then the next PW convolution is used to learn the channel correlations. Part of the features of the first PW convolution (that is, the convolution used to do channel expansion and information expansion) are directly copied and carried to the following DW convolution, and the saved computation resource through this is used to learn the features, so that the feature learning ability and expression ability of this Block can be improved (2(c)). Different types of basic convolution blocks are shown in Fig. 2.

#### TanhExp activation function

In the process of neural network modeling, nonlinear modeling capabilities are extremely important to the model. The powerful nonlinear function matching ability of the neural network is based on its internal activation function. The lightweight neural network contains only a few network layers with trainable parameters, which limits the ability to accurately simulate nonlinear functions. Effective activation functions are capable of improving the performance of these networks without sacrificing speed. The most widely used activation function is the rectified linear unit (ReLU) because of its simplicity for calculation and acceptable performance. However, in the case of non-zero mean, ReLU is the subject of the bias shift problem. Each unit that activates ReLU will cause a slight bias shift, so a series of units will cause serious situations (Zhou et al., 2020; Manurangsi & Reichman, 2018). In addition, an average far from zero will also reduce the learning speed. In the initial stage of DNN, Sigmoid and Tanh are usually adopted as activation functions due to their nonlinearity. However, the saturation of these two functions will severely limit the fitting ability of the network and may cause the gradient to disappear.

**Figure 3:**
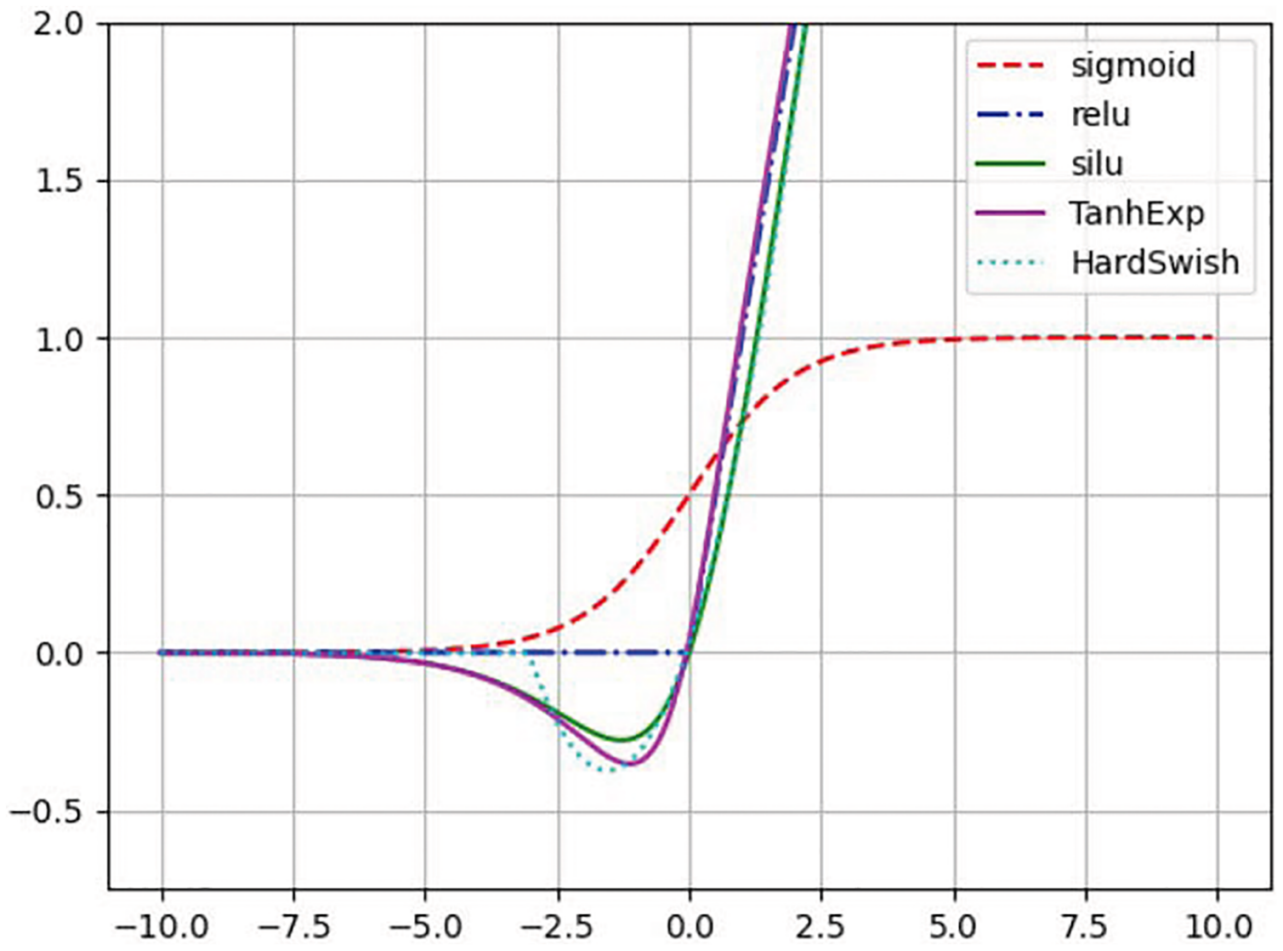
Different activation functions.

Liu & Di (2020) were the first to propose the TanhExp activation function to improve the capacity of lightweight neural network. It integrates the advantages of the ReLU and other non-segment activation functions, and therefore has a shorter calculation time. It is a continuous function with negative values, which causes the average value of activations to approach zero. Therefore, the learning process is accelerated. Its positive part is approximately linear, and the gradient close to zero is steeper, which speeds up the update of network parameters. When the input is greater than 1, the change is not greater than 0.01. In this study, the TanhExp activation function is used to replace ReLU, SiLU, HardSwish, and other activation functions.The different activation functions are shown in Fig. 3. TanhExp activation function can be defined in Eq. (1).

(1) $${\rm f}(x) = x\tanh (\mathop e\nolimits^x )$$

### Shuffle polarized self-attention

**Figure 4:**
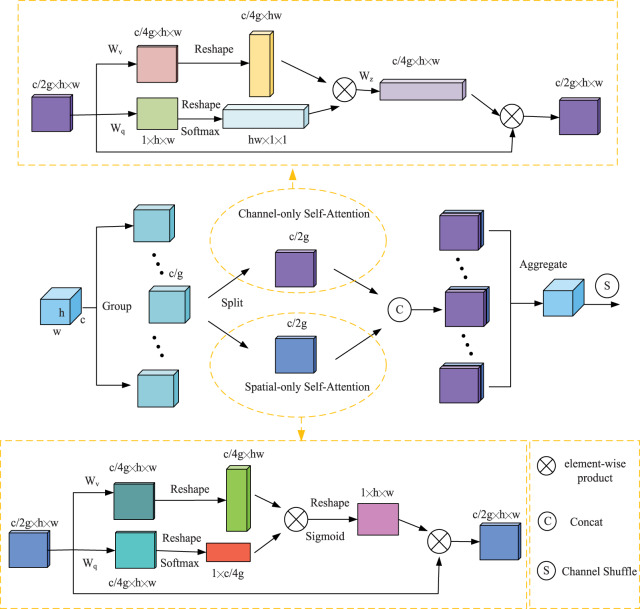
An overview of the proposed SPSA module.

The farmland scene is complex and changeable, and thus, it is difficult for the deep network to effectively extract useful target information, resulting in low detection accuracy. The attention mechanism is widely used in deep learning models by modeling the human visual perception mechanism, enabling the network to effectively extract key information and suppress irrelevant redundant information. However, detection using only local feature information is prone to false positives and missed detections. Using global contextual information allows the target to establish long-range dependencies, thus suppressing the complex background and highlighting the wheat target region from the overall image perspective. Therefore, global feature extraction is crucial for wheat detection. The self-attentive mechanism can capture the global information through the computation of global correlation. Liu et al. (2021a) employed a polarization filtering mechanism in the PSA, which is similar to optical lenses filtering light. All lateral light is reflected and refracted during photography. The function of polarization filtering is to only allow light perpendicular to the transverse direction to pass through, thereby improving the contrast of the photo. The total intensity is lost due to the filtering process, so the filtered light usually has a smaller dynamic range and requires additional boosting to recover the details of the original scene. The polarized self-attention first compresses the features in one direction and then boosts the strength range of the loss. Combining the difficulties of wheat ear detection, SPSA attention is given to adaptively select focus positions and produce a more discriminative representation of the features, this strategy introduces polarized self-attention to spatial dimension and channel dimension, and adopts Shuffle Units to combine two types of attention mechanisms effectively. The overall architecture of the SPSA module is illustrated in Fig. 4.

**Feature Groupin.** SPSA module first divides a feature tensor of one sample into multiple groups along the channel dimension. Each sub-feature gradually captures specific semantic information during the training process. Then, the attention module is used to calibrate the weight of each sub-feature according to its importance. Specifically, the sub-feature is split into two branches along the channels dimension. As illustrated in Fig. 4, one branch is adopted to produce a channel attention map by exploiting the inter-relationship of channels, focusing on “what” is a meaningful input image. The other branch is used to generate a spatial attention map by utilizing the inter-spatial relationship of features, focusing on “where” the most informative parts are, which complements the channel attention.

**Channel-only branch.** The channel attention module is mainly used to measure the importance of the channel, thereby facilitating the extraction of effective channel information. The channel attention calculation process can be expressed as follows:

(2) $${{\rm A}^{ch}}\left( X \right) = {F_{SG}}\left[ {{W_z}\left( {({\sigma _1}({W_\upsilon }(X))) \times {F_{SM}}({\sigma _2}({W_q}(X)))} \right)} \right]$$

(3) $$\mathop Z\nolimits^{ch} = \mathop A\nolimits^{ch} \mathop {\rm \odot }\nolimits^{ch} X$$

where *X* is a feature tensor of one sample, where *W*_*q*_,*W*_*v*_ and *W*_*z*_ are 1 × 1 convolution layers respectively, *σ*_1_ and *σ*_2_ are two reshape operators, and *F*_*SM*_(.) is a SoftMax operator, and “×” is the matrix dot-product operation. where “ $\mathop {\rm \odot }\nolimits^{{\rm ch}}$” is a channel-wise multiplication operator.

**Spatial-only branch.** The spatial attention module is used to highlight the feature information of the location of the target in the feature map. The spatial attention calculation process can be expressed as follows:

(4) $${A^{sp}}\left( X \right) = {F_{SG}}\left[ {{\sigma _3}\left( {{F_{SM}}({\sigma _1}({F_{GP}}({W_{\rm q}}(X)))) \times {\sigma _2}({W_{\rm v}}(X))} \right)} \right]$$

(5) $$\mathop Z\nolimits^{sp} = \mathop A\nolimits^{sp} (X)\mathop {\rm \odot }\nolimits^{sp} X$$

where *X* is a feature tensor of one sample, where *W*_*q*_ and *W*_*v*_ are standard 1 × 1 convolution layers respectively, *σ*_1_, *σ*_2_ and *σ*_3_ are three reshape operators, and *F*_*SM*_(.) is a SoftMax operator. *“F*_*GP*_(.) ” is a global pooling operator and “×” is the matrix dot-product operation. Where “ $\mathop {\rm \odot }\nolimits^{sp}$” is a channel-wise multiplication operator.

**Aggregation.** After that, all the sub-features are aggregated. Finally, the channel shuffle operator (Ma et al., 2018) is used to realize the flow of cross-group information along the channel dimension, which strengthens the mutual exchange of information.

### CIOU boundary regression loss function

**Figure 5:**
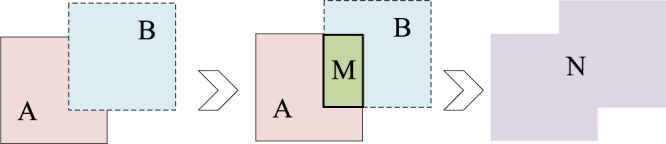
IOU diagram.

Intersection Over Union (IOU) is a common indicator used to judge the accuracy of the prediction box in target detection. It is used to measure the relative size of the overlap between the prediction box and the truth box. The larger the overlap between the prediction box and the real box is, the better the prediction effect becomes. IOU diagram is illustrated in Fig. 5.

The specific calculation is as follows:

(6) $$IOU = \displaystyle{{A \cap B} \over {A \cup B}} = \displaystyle{M \over N}$$

(7) $$IOU\_LOSS = 1 - \displaystyle{{A \cap B} \over {A \cup B}} = 1 - \displaystyle{M \over N}$$

where A and B represent the areas of predicted and real boundary boxes, M represents the intersection of two boxes, and N represents the union of two boxes. When the prediction box does not coincide with the truth box, the IOU value is 0, and the loss function cannot be differentiated and cannot reflect the position relationship between A and B. The GIOU loss function solves the problem of no overlap between the two boxes by adding the smallest rectangular box containing the detection frame and the real frame (Rezatofighi et al., 2019). The specific calculation is as follows:

(8) $$GIOU\_LOSS = 1 - IOU + \displaystyle{{\left| {C - A \cup B} \right|} \over {^C}}$$

where A and B represent the areas of predicted and real boundary boxes, C represents the smallest enclosing rectangle containing two boxes A and B.

However, when either prediction box or regression box completely contains the other or the two boxes are in horizontal and vertical directions, the GIOU loss at this time will degenerate into ordinary IOU loss and the convergence speed will be slow. On the basis of the GIOU loss function, the CIOU loss function introduces the scale information of the aspect ratio of the prediction box, and adds the distance between the center point of the prediction box and the ground truth box into the loss calculation, thereby improving the convergence speed and solution accuracy. Due to the high degree of overlap of wheat ears, CIOU loss is used as the border regression loss function. The specific calculation is as follows:

(9) $$CIOU\_LOSS = 1 - CIOU = 1 - \left( {IOU - \displaystyle{{r_2^2} \over {r_1^2}} - \alpha \nu } \right)$$

where r1 represents the diagonal distance of the rectangular box C, r2 represents the distance between the center of the real box and the predicted box. *α* is the parameter of the penalty term, *v* is used to measure similarity in aspect ratio. Where *w*^*gt*^ and *h*^*gt*^ is the width and height of the real box of the predicted target; *w* and *h* represents the width and height of the predicted target.

## Experiments

### Dataset description and analysis

To verify the effectiveness of the proposed algorithm, the Global Wheat Head Detection (GWHD) dataset was used in this study. The dataset was collected between 2016 and 2019 by nine institutions at 10 different sites, covering genotypes from Europe, North America, Australia, and Asia (David et al., 2020). There are 4,698 images with size of 1024 × 1024 pixels, and each image contains 20–70 wheat ears.

The dataset covers a range of growing environments with different climate, planting densities, and unequal row spacing. In this dataset, parameters of the sensor platform and camera shooting are also different, such as sensor size, camera focal length, ground sampling distance, half-field range along the diagonal of the image, image acquisition height and etc. It is this diversity that gives images a wide range of properties, which will help train deep learning models and enhance their universality. Some of the image characteristics of the sub-datasets composing the GWHD dataset are shown in Table 2.

**Table 2:** Some of the image characteristics of the sub-datasets composing the GWHD dataset.

Sub-dataset name	Target stage	Row spacing (cm)	Seeding density	Focal length
UTokyo_1	Post-flowering	15.0	186	10.0
UTokyo_2	Flowering	12.5	200	7.0 or 4.0
Arvalis_1	Post-flowering-Ripening	17.5	300	50.0 and 60.0
Arvalis_2	Post-flowering	17.5	300	7.7
Arvalis_3	Post-flow, erring-Ripening	17.5	300	7.7
INRAE_1	Post-flowering-Ripening	16.0	300	7.7
USask_1	n.a	30.5	250	16.0
RRes_1	n.a	n.a	350	50.0
ETHZ_1	n.a	12.5	400	35.0
NAU_1	Flowering	20.0	300 or 450	24.0
UQ_1	Flowering-Ripening	22.0	150	55.0

### Training detail

The mean of average accuracy (MAP) is a significant index to measure the efficiency of target detection. MAP is composed of Precision and Recall. The precision rate can reflect the false detection of the network model detector, and the recall rate can reflect the problem of the detector’s missed detection target. The formulas of precision rate and recall rate can be expressed as:

(10) $$\it Precision = \displaystyle{{TP} \over {TP + FP}}$$

(11) $$\mathop {{\it Recall}} = \displaystyle{{TP} \over {TP + FN}}$$

where TP means the positive sample class is retrieved, FP means the negative sample class is retrieved, FN means the positive sample class is not retrieved, and TN means that the negative sample class is not retrieved. MAP is the average of different kinds of AP, FPS is the average number of images detected per second. AP is calculated as follows:

(12) $$AP = \int_0^1 P(R)dR$$

The experiments used Python as the programming language, PyTorch as the deep learning framework and were run on the high-performance computing platform of PR4764GW. The hardware and software configurations are shown in Table 3. Before training, 2,699 wheat ear pictures were randomly selected as training set, 674 wheat ear pictures as test set. The input image pixels are 1024 × 1024, the batch size is 4, the maximum number of iterations is 100, the initial learning rate is 0.01, the weight decay rate is set to 0.0005, and the IOU threshold is set to 0.5. The cosine annealing parameter is 0.2, and the Adam optimizer is used to optimize the network parameters. In order to enhance the generalization ability of the model, methods such as scaling and mirroring the original image data, rotating within a certain angle range, saturation, and Mosaic, are adopted.

**Table 3:** Hardware and software configuration.

Name	Parameter
System	Ubuntu18.04
GPU	NVIDIA GTX 1080Ti-12G
CUDA	10.0
CUDNN	7.3.1
Pytorch	1.8.1

### Experimental results and analysis

#### Comparison study with other algorithms

**Figure 6:**
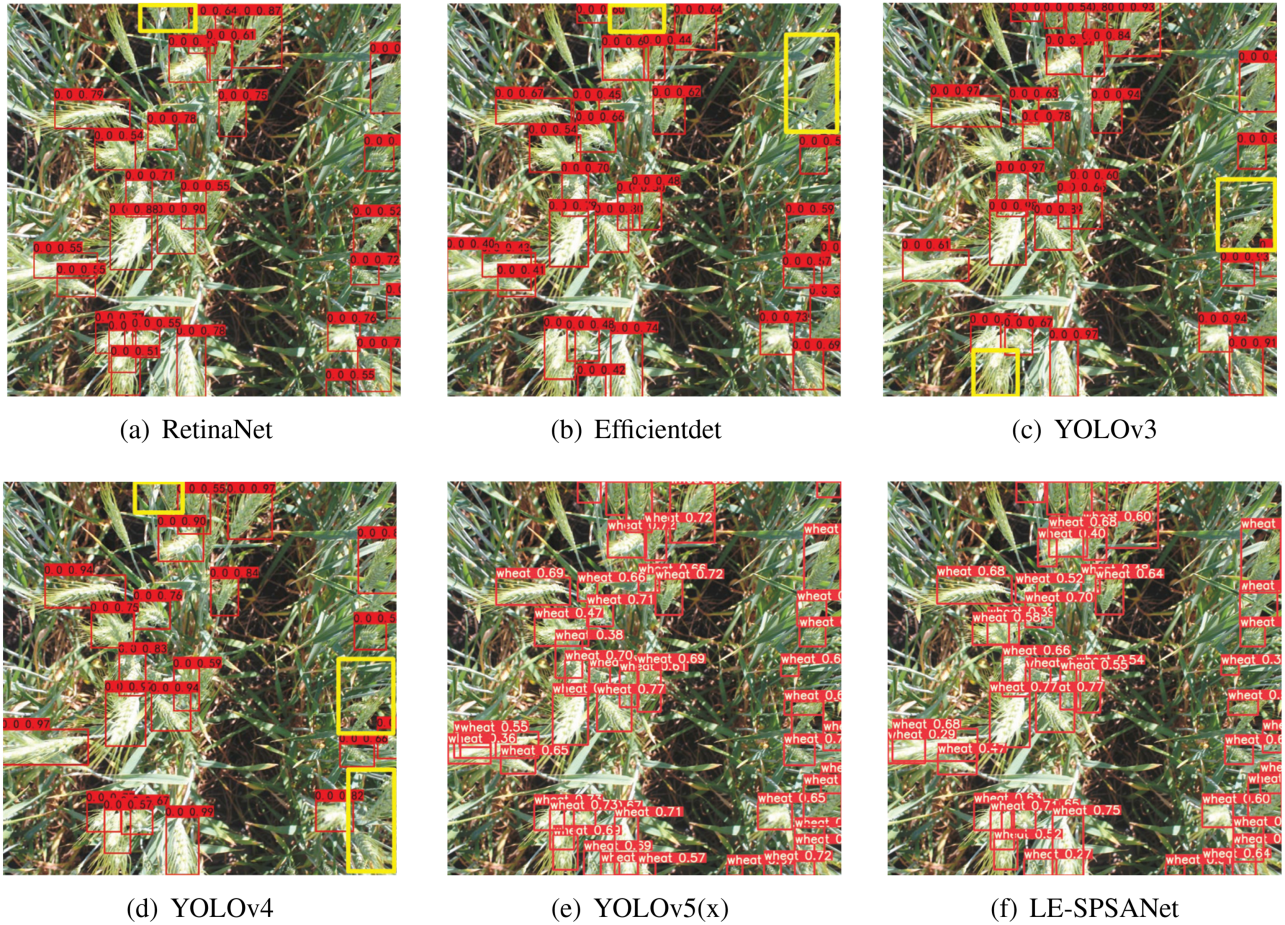
(A–F) Visualization results.

To verify the effectiveness of our designed deep neural network for the wheat detection task, we tested the performance of some mainstream object detection models on the wheat detection task, for instance, RetinaNet (Lin et al., 2017), Efficientdet (Tan, Pang & Le, 2020), YOLOv3 (Redmon & Farhadi, 2018), YOLOv4 (Bochkovskiy, Wang & Liao, 2020), YOLOv5(x) (Ultralytics, 2020). The visualization results are shown in Fig. 6.

As the visualization results show, the algorithms (a)–(d) can easily detect most of the wheat targets, yet the wheat targets in the yellow box with high density and relatively complex background cannot be effectively detected. Meanwhile, YOLOv5 and LE-SPSANet show better detection results.

To further evaluate the performance of the proposed method in this paper. The model capacity, detection speed, and mAP of the above algorithms are compared in this paper. The experimental results are shown in Table 4.

**Table 4:** The results of each detection algorithm.

Algorithm	Input	Model size (MB)	FPS (frames/s)	MAP%
EfficientDet (D0)	512*512	15.07	14	85.5
YOLOv4	416*416	244.29	14	91.2
RetinaNet	416*416	138.91	16	91.9
YOLOv3	416*416	235.04	39	92.1
YOLOv5(x)	1024*1024	177.5	10	93.6
LE-SPSANet	1024*1024	9.0	25	94.4

Efficientdet and RetinaNet are excellent anchor-based object detectors, which need to extract candidate anchors before making predictions. However, due to the complex background, dense target, and large amount of overlaps of the wheat spike, they are likely to miss small and dense objects, leading to relatively low precision of only 85.5% and 91.9% MAP separately.

YOLOv3 is a classic one-stage object detector which divides the entire image evenly into multiple grids and directly predicts N anchor sizes for each grid. However, it achieves high detection accuracy at the cost of much larger capacity, which is detrimental to its application in resource-limited scenarios. It is worth noting that YOLOv3 shows higher detection performance compared to YOLOv4. Through the visual detection results (6(c), 6(d)), we found that YOLOV3 has fewer missed detections for dense targets than YOLOv4 in the GWHD dataset and therefore has higher accuracy.

YOLOv4 and YOLOv5 are both mainstream object detectors which perform optimization in many aspects including activation functions, backbone networks, loss functions and etc. in the field of CNN in recent years. However, due to the denseness of the objects in the GWHD dataset, the detection speeds are 14 fps and 10 fps, respectively.

LE-SPSANet is also a one-stage object detection network, but compared to the YOLO series, the speed can be as high as 25 fps, which is 15fps faster than YOLOv5(x). In terms of accuracy, our network is more suitable for detecting dense objects and multi-scale objects, which is 8.9% higher than Efficientdet. LE-SPSANet’s MAP is 94.4%, which is 0.8% more than YOLOv5(x), but its speed is 1.5 times faster and is only 5.07% of it. Therefore, it can be said that LE-SPSANet has reached a balance between speed, accuracy, and model size.

#### Comparison of ablation results

**Figure 7:**
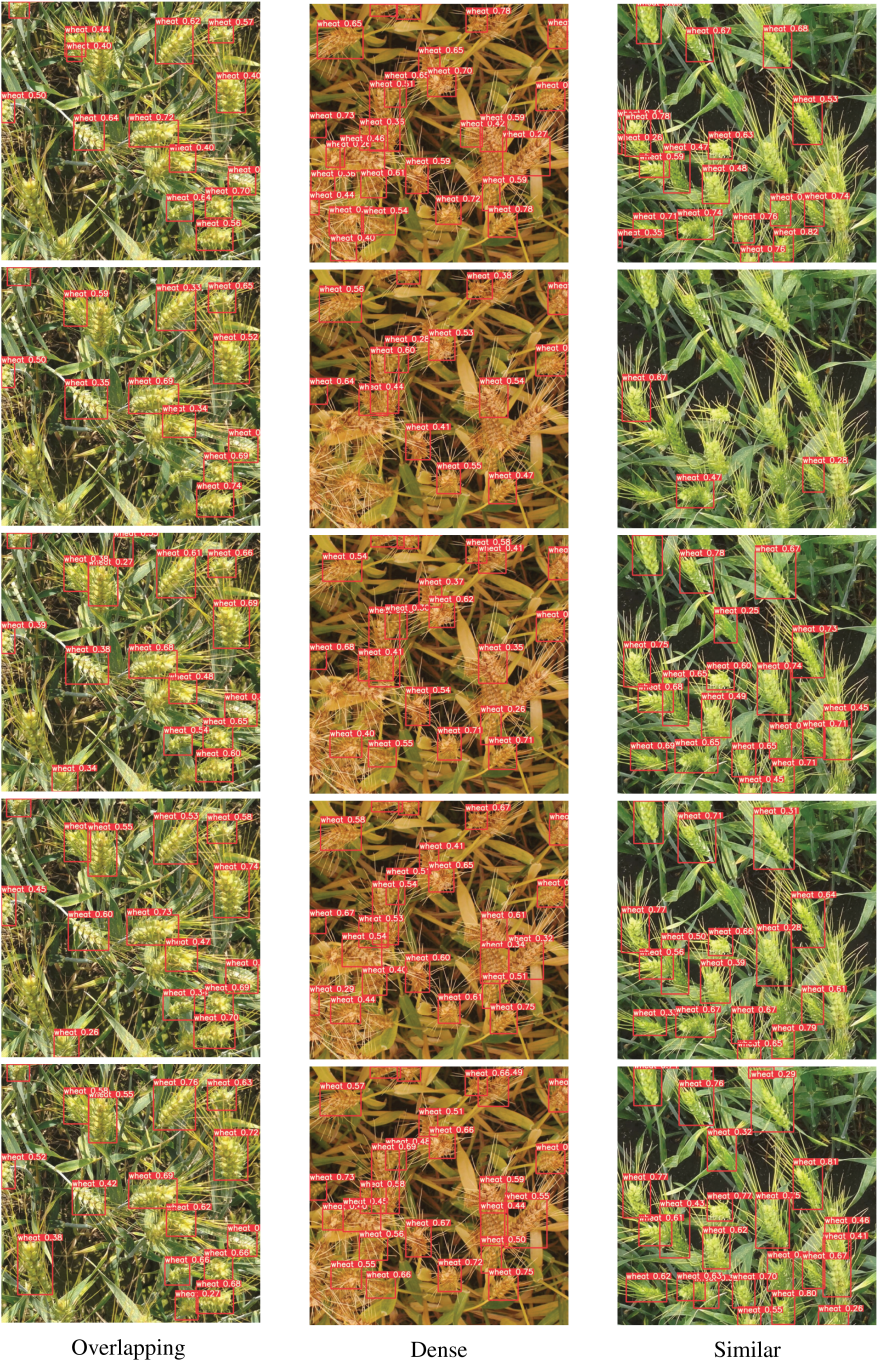
Comparison of the effects of different modules on wheat ear detection. The three pictures (respectively represent high overlap, densely packed wheat ears, and similar backgrounds) in each row are a group, namely Group1–Group5.

In the context of complex deep learning networks, an ablation study is used to describe the performance of the network after removing some modules, which is beneficial to better understanding the influence of different modules. In order to determine the influence of each module on network performance, this paper sets five groups of experiments for training respectively. Group1 is the YOLOv5(x) model and groups 2 to 5 are algorithms with improved modules, where ‘+’ indicates that the improved module is included and “−” indicates that the improved module is excluded. Experimental results are shown in Table 5 below. Three representative wheat ear pictures are selected from the test dataset, and the visual display of the ablation experiment is performed with regard to difficulties of high overlap, dense wheat ears, and similar background. The visualization results are shown in Fig. 7.

**Table 5:** Comparison of ablation result.

	AsymmNet	CIOU	SPSA	TanhExp	Model size (MB)	FPS (frames/s)	MAP%
1	–	–	–	–	177.5	10	93.6
2	+	–	–	–	12.9	23	91.7
3	+	+	–	–	12.9	27	93.5
4	+	+	+	–	9.0	21	94.3
5	+	+	+	+	9.0	25	94.4

In the first group, YOLOv5 shows a good detection effect on most wheat ear targets at the cost of detection speed and model capacity. However, the ability to detect wheat ear regions with more target overlaps and higher background similarity needs to be improved.

After the backbone network is used with AsymmNet in the second group, the total parameters decrease to 7.2% of YOLOv5, and the operation speed increases to 23 frames/s at the expense of accuracy. AsymmNet backbone network is lighter and more efficient than CSPDarkNet53. Although the introduction of the lightweight backbone network inevitably causes a decrease in detection accuracy, it ensures that the whole detector proposed is fast and the number of parameters of the model is small.

In the third group, CIOU is used as the boundary regression loss function, which greatly improves the detection accuracy of the wheat ear region with more overlaps under the condition of the same parameter amount, and also improves the detection speed. From the visualized results, Group3 has improved the detection effect for dense overlaps wheat ears region compared with Group2, and the missed detection of wheat ears has been improved. The detection effect for the wheat ears region with the high similarity between wheat ears and wheat leaves is improved significantly. This also fully illustrates the effectiveness of the CIOU bounding box regression loss function for detecting dense small targets.

In the fourth group, SPSA module is added, and the SPSA attention mechanism improves the sensitivity of the network model to key features, increases the weight of the wheat features in the network, eliminates irrelevant redundant features, and improves the robustness of the network. It not only improves the accuracy of the overall prediction box but also reduces the capacity of the model. In terms of visualization, Group4 has further improved the detection of dense and overlapping wheat regions compared to Group3, especially the regions with high overlap.

In the fifth group, we use the TanhExp activation function instead of ReLU, SiLU, HardSwish, or other activation functions. It reduces the training time and improves the detection speed without changing the accuracy and model capacity. In terms of its detection visualization results, it has a better detection effect for wheat with dense and complex background. This experiment uses the LE-SPSANet algorithm. In conclusion, the LE-SPSANet algorithm proposed in this paper has great performance and is more suitable for target detection of wheat ears.

## Conclusions

To improve the accuracy of wheat spike detection in complex farmland scenarios and facilitate deployment on edge equipment, this paper combines the lightweight and efficient AsymmNet backbone network to reduce model capacity, improve feature extraction capabilities, and enrich feature information. In order to highlight the key feature information and weaken the general redundant information, SPSA is given to filter and weight the feature vector. CIOU loss is used as the border regression loss, which considered the center distance, overlap rate, and aspect ratio between the prediction frame and the truth frame. training time was greatly reduced by using the TanhExp activation function. The experimental results show that the LE-SPSANet algorithm proposed in this paper can effectively detect wheat spike targets, and the total parameter is only 5.07% of YOLOv5(x), the detection speed is increased by 15 frames/s, the model complexity is reduced by 23.1 times, and the detection accuracy is improved to 94.4%. The next step of this paper will consider optimizing the network structure, enhancing the generalization ability of the model, and speeding up the network detection speed while ensuring the improvement of detection accuracy.

## Additional Information and Declarations

### Data Availability

The following information was supplied regarding data availability: The code is available at GitHub: https://github.com/liuyundong-2020/LE-SPSANet. The data is available at Zenodo: DAVID, Etienne. (2020). Global Wheat Head Dataset-2020 challenge version [Data set]. In Plant Phenomics (Version 4). Zenodo. https://doi.org/10.5281/zenodo.4298502.
